# Chromosome-level genome assembly of *Solanum pimpinellifolium*

**DOI:** 10.1038/s41597-024-03442-6

**Published:** 2024-06-04

**Authors:** Hongyu Han, Xiuhong Li, Tianze Li, Qian Chen, Jiuhai Zhao, Huawei Zhai, Lei Deng, Xianwen Meng, Chuanyou Li

**Affiliations:** 1 College of Agronomy, Shandong Agricultural University, Tai’an, 271018 China; 2 Taishan Academy of Tomato Innovation, Shandong Agricultural University, Tai’an, 271018 China; 3 College of Horticulture Science and Engineering, Shandong Agricultural University, Tai’an, 271018 China; 4 College of Life Sciences, Shandong Agricultural University, Tai’an, 271018 China; 5 State Key Laboratory of Black Soils Conservation and Utilization, Northeast Institute of Geography and Agroecology, Chinese Academy of Sciences, Changchun, 130102 China; 6 Key Laboratory of Soybean Molecular Design Breeding, Northeast Institute of Geography and Agroecology, Chinese Academy of Sciences, Changchun, 130102 China

**Keywords:** Genomics, Genome informatics

## Abstract

*Solanum pimpinellifolium*, the closest wild relative of the domesticated tomato, has high potential for use in breeding programs aimed at developing multi-pathogen resistance and quality improvement. We generated a chromosome-level genome assembly of *S. pimpinellifolium* LA1589, with a size of 833 Mb and a contig N50 of 31 Mb. We anchored 98.80% of the contigs into 12 pseudo-chromosomes, and identified 74.47% of the sequences as repetitive sequences. The genome evaluation revealed BUSCO and LAI score of 98.3% and 14.49, respectively, indicating high quality of this assembly. A total of 41,449 protein-coding genes were predicted in the genome, of which 89.17% were functionally annotated. This high-quality genome assembly serves as a valuable resource for accelerating the biological discovery and molecular breeding of this important horticultural crop.

## Background & Summary

Tomato (*Solanum lycopersicum*) is one of the most valuable vegetable crops worldwide. It also serves as a classic model system for studying plant-pathogen interactions and fruit development^1,2^. Fruit size increased gradually during tomato domestication; however, continued selection reduced the genetic diversity, causing the loss of multiple disease resistance in cultivated species^3,4^. Thus, wild tomato species have been frequently used as important germplasm donors in modern tomato breeding programs^5,6^. *S. pimpinellifolium*, the wild progenitor of the cultivated tomato^7^, possesses genes that confer resistance to biotic and abiotic stresses^8,9^; for example, *Sm* from *S. pimpinellifolium* PI79532 confers high resistance against gray leaf spot in tomato^10^; the *I* gene, also derived from PI79532, confers resistance against *Fusarium oxysporum* f. sp. *lycopersici* races 1^11^; *Rx4* from *S. pimpinellifolium* PI128216 confers hypersensitive resistance to bacterial spot race T3^12^; and *Ph-3* derived from *S. pimpinellifolium* L3708, confers resistance to *Phytophthora infestans*^13^. These findings indicate the huge potential of *S. pimpinellifolium* for use in breeding programs to develop disease-resistant varieties.

**Fig. 1:**
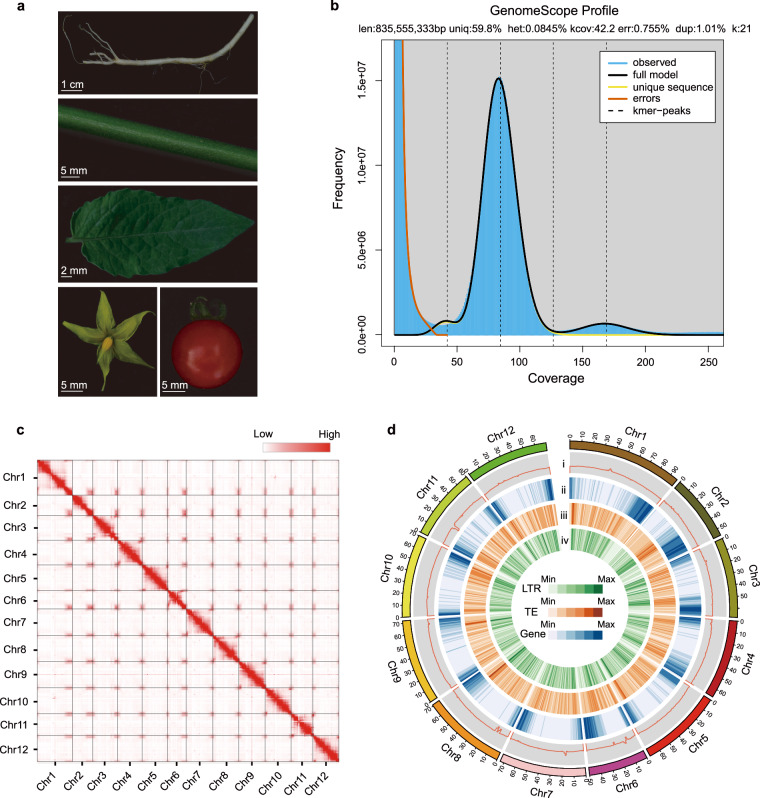
Overview of the *S. pimpinellifolium* LA1589 genome assembly and features. (**a**) Morphology of the root, stem, leaf, flower, and fruit of LA1589. (**b**) Genomescope profile for 21-mers based on Illumina short-reads. (**c**) Hi-C contact map the chromosome-level assembly of LA1589. (**d**) Genome features of LA1589. For the circos map, the tracks from outside to inside are: (i) GC content (%); (ii) density of protein-coding genes; (iii) TE density; (iv) LTR density.

Whole-genome sequencing improves molecular breeding because high-quality plant genomes facilitate the identification of genetic diversity among different germplasms^14–17^. Currently, chromosome-level genome assemblies are available for the cultivated tomatoes, such as *S. lycopersicum* cv. M82^18^ and Heinz 1706^19,20^, and wild tomatoes, such as *S. pennellii* LA0716^21^ and *S. galapagense* LA0436^22^. All these genome assemblies provide favorable support for the discovery of causal genetic variations underlying the major tomato traits based on comparative genomic analysis. *S. pimpinellifolium* LA1589 is a wild-type tomato accession with small, red, round fruits (Fig. 1a) that is widely used for trait mapping^23–26^. Particularly, the well-established introgression line population from cross of *S. lycopersicum* cv. E6203 and LA1589 represents one of the widest crosses and serves as an important source for scientists and breeders^27^. Although the draft genome assembly of this accession was published 10 years ago^28^, a chromosome-level genome sequence has not yet been published, and thus the vast majority of sequence variations are poorly characterized and their impact on important traits are largely hidden.

In this study, we assembled the chromosome-level genome of *S. pimpinellifolium* using a combination of short-read sequencing, PacBio sequencing, Hi-C scaffolding, and Bionano optical mapping technologies. The resulting assembly has a total length of 833 Mb, with a contig N50 of 31 Mb, a complete BUSCO value of 98.3%, and a high LAI score of 14.49. The high-quality *S. pimpinellifolium* genome assembled in this study provides a valuable genetic resource for future efforts to study tomato domestication and promote genome-scale breeding.

## Methods

### Library construction and genome sequencing

The seeds of *S. pimpinellifolium* LA1589 were acquired from TGRC (https://tgrc.ucdavis.edu/) and planted in the greenhouse at the Institute of Genetics and Developmental Biology, Chinese Academy of Sciences (Beijing, China). Total genomic DNA was extracted from fresh young leaves using the CTAB method^29^. A Pacific Biosciences (PacBio) SMRT library was constructed from high molecular weight DNA following the standard SMRTbell library preparation protocol. A total of five SMRT cells were run on the PacBio Sequel system. For short-read sequencing, the paired-end libraries with a 350-bp insert length were constructed and sequenced using the BGISEQ-500 platform. A high-throughput chromosome conformation capture (Hi-C) library was prepared following the proximo Hi-C plant protocol (Phase Genomics) and sequenced using an Illumina NovaSeq. 6000 platform with the paired-end mode. For BioNano optical mapping, genomic DNA was isolated using a BioNano Plant Tissue DNA Isolation Kit. Labelled genomic DNA was then loaded onto the BioNano Saphyr System.

### Genome survey

The k-mer frequency method was employed to estimate the genome size. The short-read sequencing produced 104.7 Gb of clean data after filtering out low-quality reads. Jellyfish v2.2.10^30^ (count -C -m 21; histo -h 40000) was used to compute a histogram of 21 k-mer frequencies. The heterozygosity level was calculated using GenomeScope v1.0^31^. As a result, the estimated genome scale of *S. pimpinellifolium* was 835.55 Mb, with a heterozygosity rate of 0.08% (Fig. 1b).

### Genome assembly and quality assessment

The PacBio sequencing produced 282.3 Gb long reads. Canu v1.8^32^ (genomeSize = 800 m minOverlapLength = 600 minReadLength = 1000) was used to assemble PacBio subreads to PacBio contigs. BioNano optical maps were assembled into consensus physical maps using BioNano Solve v3.1 (https://bionanogenomics.com/). HERA v1.0^33^ was used to extend and connect the contigs, and to fill in gaps in the BioNano hybrid scaffolds. The 128.5 Gb Hi-C reads were mapped to the scaffolds with Bowtie2^34^. Then, HiC-Pro^35^ was employed to align the pair-end reads and Juicebox^36^ was used to build the interaction map (Fig. 1c). The scaffolds were further clustered and assigned to different chromosomes. To increase the accuracy of the assembly, Illumina short reads were mapped to genome using BWA v0.7.15^37^. Next, the genome was corrected using Pilon v1.24^38^, and three rounds of genome correction were performed. The 833.19-Mb final assembly had a contig N50 length of 31.2 Mb, and approximately 98.87% of the assembled sequence was anchored onto 12 pseudo-chromosomes (Fig. 1d), and showed a greater improvement compared to the previous version of LA1589 genome assembly released in 2012. Moreover, it was also very outstanding when compared with the reference assemblies of *S. pennellii* LA0716 and *S. lycopersicum* cv. Heinz 1706 (Table 1).

**Table 1:** Comparison of tomato genome assemblies.

	LA1589 (2023)	LA1589 (2012)	LA0716 (2014)	Heinz 1706 (2019)
Contig N50 (bp)	31,220,755	5,710	1,741,129	6,007,830
Contig N60 (bp)	26,821,871	4,085	1,353,889	4,652,653
Contig N70 (bp)	15,037,013	2,682	1,059,177	3,851,369
Contig N80 (bp)	10,225,340	1,568	763,066	2,733,934
Contig N90 (bp)	7,509,232	765	437,042	1,566,229
Maximum contig length (bp)	60,886,717	80,806	10,011,355	26,291,688
Number of contigs	272	309,695	4,579	448
Total assembled length (Mb)	833	688	942	782
Anchored to chromosomes (Mb)	823	—	926	772
Complete BUSCOs (%)	98.3	71.6	97.9	97.9
Number of gene models	41,449	34,727	44,965	34,075

The completeness of the genome was evaluated using BUSCO (Benchmarking Universal Single-Copy Orthologs) v5.4.5^39^ program with the Solanales odb10 dataset, revealing 98.3% of Solanaceae BUSCOs were captured in this assembly (Table 2). Furthermore, the contiguity of the genome was evaluated by calculating LTR Assembly Index (LAI)^40^ using LTR_retriever v2.9.9^41^ with default parameters. The LAI value of the genome assembly was 14.49. Collectively, these results indicate a high quality of the *S. pimpinellifolium* genome assembly.

**Table 2:** BUSCO analysis of the genome assembly.

	Number of BUSCOs	Percent (%)
Complete	5,849	98.3
Complete and single-copy	5,741	96.5
Complete and duplicated	108	1.8
Fragmented	12	0.2
Missing	89	1.5
Total	5,950	—

### Repeat annotation

**Fig. 2:**
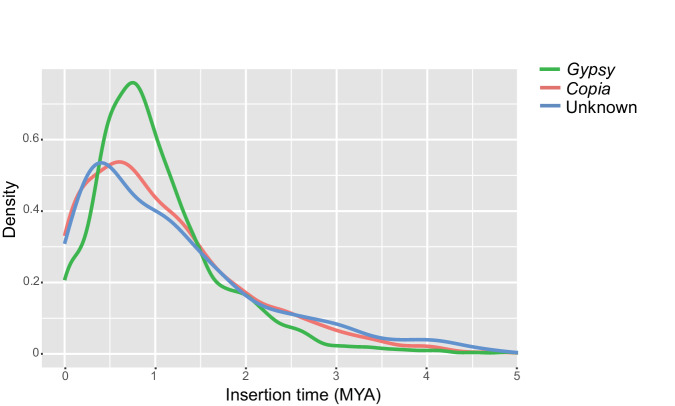
Overall insertion time distribution of LTR elements in the *S. pimpinellifolium* genome.

The transposable element (TE) libraries were obtained by running the EDTA pipeline^42^. In addition, short interspersed nuclear element (SINE) candidates were predicted by the SINE-Finder program v1.0^43^ and integrated into the TE library. RepeatMasker v4.0.7^44^ was used for homologous repeat identification by running against the consensus TE library. Approximately 74.47% of the genome was composed of repetitive sequences (Table 3). LTRs represented the largest proportion (47.45%) of repetitive elements in the genome, of which *Gypsy* (28.12%) was the most abundant. The insertion time of long terminal repeat (LTR) retrotransposons was estimated as described previously^45^. In brief, the 5′ and 3′ end terminal repeat sequences of each LTR were extracted and aligned using MUSCLE v3.8.1551^46^. Next, the insertion time of LTR was calculated by *T* = *K/2r*, where *K* is the divergence rate and *r* is the neutral mutation rate. The results showed that the main burst of *Gypsy* elements occurred about 0.75 million years ago (MYA), whereas the main burst of *Copia* elements occurred about 0.6 MYA (Fig. 2), indicating that the amplification of *Gypsy* elements occurred prior to that of *Copia* elements and that *Gypsy* expansion had a major effect on the *S. pimpinellifolium* genome expansion.

**Table 3:** Classification of transposable elements in the *S. pimpinellifolium* genome.

	Number	Coverage (Mb)	Fraction of genome (%)
LTR/Gypsy	174,466	172,516,542	20.94
LTR/Copia	67,848	43,662,565	5.3
LTR/unknown	127,392	74,888,856	9.09
SINE	48,430	10,955,007	1.33
LINE	1,660	611,688	0.07
Total class I	419,796	302,634,658	36.74
hAT	15,517	7,036,940	0.85
CACTA	121,326	71,332,210	8.66
PIF-Harbinger	29,904	17,402,335	2.11
Mutator	165,516	101,398,344	12.31
Tc1/Mariner	22,837	9,006,747	1.09
MITE	30,955	6,299,938	0.76
Helitron	225,547	98,338,894	11.94
Total class II	611,602	310,815,408	37.73
Total TEs	1,031,398	613,450,066	74.47

### Gene prediction and annotation

Protein coding genes (PCGs) in the *S. pimpinellifolium* genome were annotated using the MAKER pipeline v3.01.04^47^. Nucleotide and protein sequences from Heinz 1706 v4.0 (https://solgenomics.net/) were used as queries for homology-based predictions. Ab initio gene prediction methods used within MAKER included SNAP v2006-07-28^48^ and AUGUSTUS v2.5.5^49^. Homology-based and ab initio-based gene prediction resulted in the identification of 41,449 PCGs, which was 6,722 more genes than in the previous version of the genome. Functional annotation of the PCGs was performed using Hayai-Annotation Plants v1.0.2^50^ and KOBAS^51^. The predicted protein sequences were searched against the InterPro^52^, Swiss-Prot^53^, and NR (https://www.ncbi.nlm.nih.gov/protein) databases. In total, 36,960 (89.17%) genes were assigned specific functions (Table 4). Orthologous genes were identified using MCScanX^54^ and OrthMCL v2.0.9^55^. A total of 29,542 LA1589/Heinz 1706 orthologs were identified.

**Table 4:** Function annotation of predicted protein-coding genes.

	Number	Percent of all genes (%)
Total genes	41,449	—
GO	8,185	19.75
KEGG	33,390	80.56
InterPro	33,464	80.74
Swiss-Prot	25,993	62.71
NR	35952	86.74
Total annotated	36960	89.17

The first category of non-coding genes, tRNAs, were annotated by tRNAscan-SE v2.0.3^56^. rRNAs were annotated by RNAmmer v1.2^57^. miRNAs and snRNAs were predicted by the cmscan module in INFERNAL v1.1.2^58^ (--cut_ga --rfam --fmt 2) with searches against the Rfam database v14.9^59^. In total, four types of noncoding RNA, including 1073 tRNAs, 698 rRNAs, 582 snRNAs, and 405 miRNAs were identified from the genome.

## Data Records

The raw sequencing data generated in this study have been deposited in NCBI Sequence Read Archive with accession number SRP471177^60^ and in NGDC Genome Sequence Archive with the accession number CRA012446^61^. The final genome assembly has been deposited in GenBank under accession GCA_034621305.1^62^. The genome annotations are available from the Figshare^63^.

## Technical Validation

The quality of the *S. pimpinellifolium* assembly was evaluated using three approaches. First, the completeness of the genome assembly was assessed using BUSCO v5.4.5 and 98.30% of the BUSCO genes were complete. Then, the assembly continuity was determined by analyzing the LTR Assembly Index (LAI). The LAI score (14.49) met the quality standard for reference genomes. Additionally, for the assessment of the correctness of the genome assembly, we re-aligned clean Illumina DNA sequencing data against the assembly using BWA v0.7.15, and 99.77% reads could be successfully mapped. All these statistics indicated that this *S. pimpinellifolium* genome is of high accuracy and completeness.

## Data Availability

All pipeline and software used in this study were performed to data analysis according to the manuals and protocols. The parameters and the version of the software are described in the Methods section. If no detailed parameters are mentioned for a software, the default parameters were used.
